# 

**Published:** 1887-06

**Authors:** 


CONTENTS:

ARTICLE I.—The Odontological Society of Great Britain.......... 49
" II.—Odonto-Chirurgical Society of Scotland.......... 76
The American Dental Association.......... 87
Virginia State Dental Association.......... 88
Southern Dental Association.......... 89
American Dental Association.......... 89
The National Association of Dental Examiners.......... 89

EDITORIAL, ETC.

A Romantic History Connected with a Prominent Dentist.......... 90

MONTHLY SUMMARY.

Elements.......... 92

Artificial Teeth.......... 93

Laboratory Hints.......... 94

Students' Society.......... 95

Amalgam.......... 96

